# Discovery of SARS-CoV-2 3CL^Pro^ Peptidomimetic Inhibitors through the Catalytic Dyad Histidine-Specific Protein–Ligand Interactions

**DOI:** 10.3390/ijms23042392

**Published:** 2022-02-21

**Authors:** Yaxin Wang, Binghong Xu, Sen Ma, Hao Wang, Luqing Shang, Cheng Zhu, Sheng Ye

**Affiliations:** 1 Tianjin Key Laboratory of Function and Application of Biological Macromolecular Structures, School of Life Sciences, Tianjin University, Tianjin 300072, China; wangyaxin@tju.edu.cn (Y.W.); binghong_xu@tju.edu.cn (B.X.); masen@tju.edu.cn (S.M.); 2 KLMDASR of Tianjin and Drug Discovery Center for Infectious Disease, State Key Laboratory of Medicinal Chemical Biology, College of Pharmacy, Nankai University, Tianjin 300353, China; 1001110228@pku.edu.cn (H.W.); tju_biodesign@163.com (L.S.)

**Keywords:** SARS-CoV-2, covalent inhibitor, molecular dynamics simulations, molecule design

## Abstract

As the etiological agent for the coronavirus disease 2019, severe acute respiratory syndrome coronavirus 2 (SARS-CoV-2) challenges the ongoing efforts of vaccine development and drug design. Due to the accumulating cases of breakthrough infections, there are urgent needs for broad-spectrum antiviral medicines. Here, we designed and examined five new tetrapeptidomimetic anti-SARS-CoV-2 inhibitors targeting the 3C-Like protease (3CL^Pro^), which is highly conserved among coronaviruses and essential for viral replications. We significantly improved the efficacy of a ketoamide lead compound based on high-resolution co-crystal structures, all-atom simulations, and binding energy calculations. The inhibitors successfully engaged the catalytic dyad histidine residue (H41) of 3CLPro as designed, and they exhibited nanomolar inhibitory capacity as well as mitigated the viral loads of SARS-CoV-2 in cellular assays. As a widely applicable design principle, our results revealed that the potencies of 3CL^Pro^-specific drug candidates were determined by the interplay between 3CL^Pro^ H41 residue and the peptidomimetic inhibitors.

## 1. Introduction

Rapidly evolving pathogens, such as coronaviruses, pose major threats to global public health [1,2]. Following the 2002–2003 severe acute respiratory syndrome (SARS) and the 2012 Middle East respiratory syndrome (MERS) epidemics, the outbreak of SARS-CoV-2 in 2019 has caused more than 350 million infections thus far and took >5 million lives worldwide [3,4]. As the pathogen of coronavirus disease 2019 (COVID-19), SARS-CoV-2 constantly mutates to evade the recognition of the human immune system, mainly through modifications at the viral surface spike proteins. For instance, the delta and omicron variants (also known as the B.1.617.2 and B.1.1.529 lineages) emerged as the variants of concern and dominated the viral populations in different areas. The delta variant was resistant to neutralization mediated by monoclonal antibodies (e.g., bamlanivimab) or by sera from vaccinated individuals [5]. Hence, there are urgent needs to develop broadly neutralizing antibodies or broad-spectrum antiviral agents against the conserved viral targets to end the COVID-19 pandemic.

**Figure 1:**
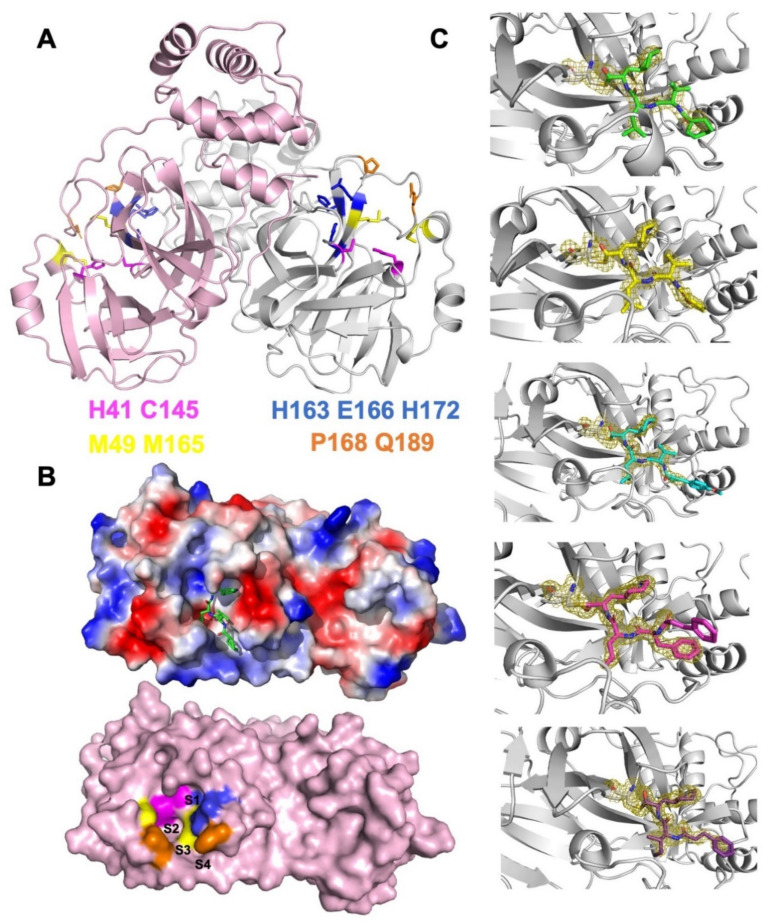
SARS-CoV-2 3CL^Pro^ as a validated antiviral drug target. (**A**) Structural model of 3CL^Pro^ with key residues at the S1–S4 pockets shown as sticks (magenta: catalytic dyad; blue: S1; yellow: S2; orange: S3 and S4). (**B**) Protein surface representation of 3CL^Pro^ protomer. The lower panel indicates the locations of the S1, S2, S3, and S4 pockets, and the upper panel shows the electrostatic potentials (blue: positively charged; red: negatively charged) with a model of ligand fitted into the binding pocket. (**C**) The crystal structures of the designed TPMs binding to 3CL^Pro^ via C145–formyl covalent bonds. 2Fo–Fc electron densities of each TPM ligand and the C145 residue are highlighted as meshes (contoured at 1.5 σ).

Among the 29 viral proteins encoded by the SARS-CoV-2 RNA genome, the 3C-Like protease (3CL^Pro^, otherwise known as main protease) is indispensable for coronavirus replication [6]. It possesses > 96% sequence identity among global coronavirus strains (Figure S1), while it distinguishes itself from the host-cell proteases by its exclusive cleave site of Leu-Gln↓ (Ser, Ala, and Gly) (↓ denotes the 3CL^Pro^ cleavage site). SARS-CoV-2 3CL^Pro^ features a dyad catalytic center composed of the highly conserved histidine and cysteine residues (H41 and C145, Figure 1A), which are responsible for the proteolytic processing of polyprotein precursors pp1a and pp1ab [7]. When applied at different stages of the virus life cycle (entry, replication, or maturation), inhibitors against 3CL^Pro^ specifically suppressed the replication of coronavirus. Despite the intense efforts of designing 3CL^Pro^ specific inhibitors and optimizing the overall binding capacities, however, clinically effective treatments against the 3CL^Pro^ viral target remain elusive [8,9].

More than twenty SARS-CoV-2 3CL^Pro^ specific inhibitors have been discovered since 2020, either by the computer-aided design of structurally novel candidates or by high-throughput screening of large compound libraries [10,11,12,13,14,15,16]. Among them, the covalent inhibitors interact with C145 of the catalytic dyad for blocking the nucleophile cleavage of peptide bonds. The successful application of electrophilic reactive groups (e.g., aldehyde, pyrogallol, and acrylamide) as warheads in covalent inhibitors has led to a sub-nanomolar binding affinity and extended duration of action [11,17]. As an oral medicine for SARS-CoV-2, the co-administration of PF07321332 (3CL^Pro^ inhibitor) and Ritonavir (HIV protease inhibitor) reduced overall hospitalizations by 89% in the phase II/III trials [18,19]. The majority of the research efforts, however, have been devoted to enhancing the linkage between C145 and small molecules, while the structural or functional impacts of the other catalytic residue, H41, remain largely overlooked. Hence, in the current study, we investigated key factors that determine the efficacies of a series of peptidomimetic 3CL^Pro^ inhibitors based on co-crystal structures (Figure 1C), molecular dynamics simulations, and in vitro as well as ex vivo cellular assays. We found their specific interactions with H41 play important roles in their ability to suppress the SARS-CoV-2 3CL^Pro^ activities. 

## 2. Results

### 2.1. Structural-Based Rational Design of TPM Inhibitors

**Figure 2:**
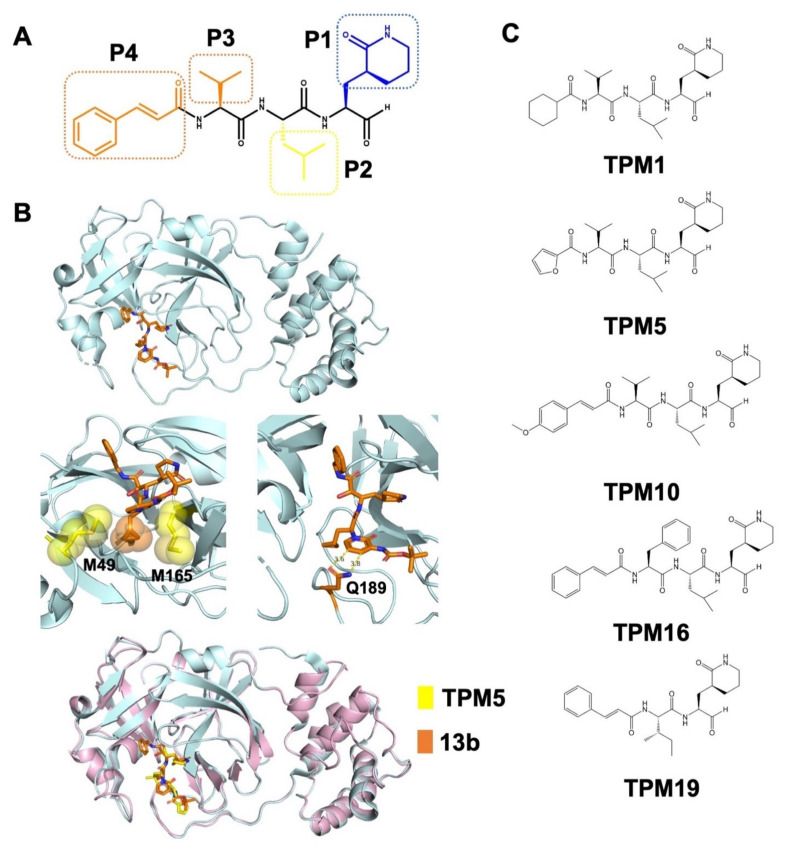
Rational design of peptidomimetic molecules as SARS-CoV-2 3CL^Pro^ inhibitors. (**A**) Scheme of the TPM design with the P1–P4 chemical groups labeled (the chemical structural formula of TPM16 was shown as an example). (**B**) Structural model of the lead compound **13b** bound with 3CL^Pro^ (PDB ID: 6Y2F). The interactions between the P2 cyclopropyl group and the M49/M165 residues and the interactions between the P3 pyridone ring and the Q189 residue are illustrated in the middle panel (van der Waals interactions represented as spheres). **13b** and TPM bound 3CL^Pro^ with similar modes in the superimposed model (lower panel). (**C**) Chemical structures of TPM1, 5, 10, 16, and 19.

**Figure 3:**
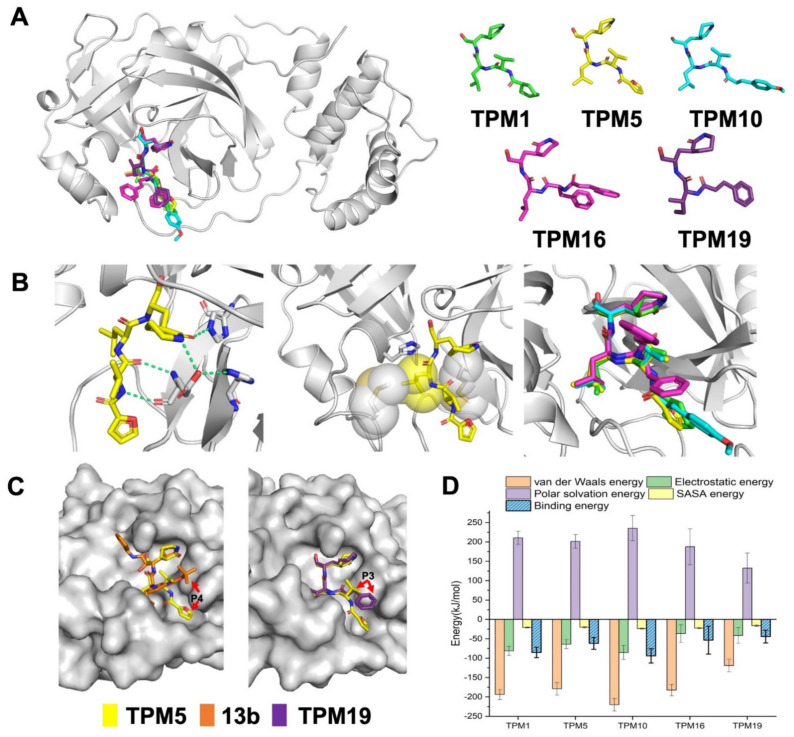
Crystal structures of the 3CL^Pro^–TPM complexes. (**A**) 3D representation of five TPMs and their overall binding modes. (**B**) The P1 δ-lactam ring formed polar interactions (green, dash line) with S1 residues (left), P2 isopropyl group stacked with S2 hydrophobic residues (middle), and the alignment of isopropyl groups from different TPMs (right). (**C**) The P3/P4 group of TPM5 bound into the S3/S4 groove in comparison to that of **13b** or TPM19. (**D**) The binding energies between 3CL^Pro^ and TPMs from all-atom simulations.

The binding pocket of SARS-CoV-2 3CL^Pro^ can be subdivided into four sub-pockets (S1–S4, Figure 1B). We optimized a recently published lead compound **13b** based on its complex structure with 3CL^Pro^ (PDB ID: 6Y2F) [16]. We adopted a tetrapeptidomimetic scheme for the inhibitor design (denoted as tetrapeptidomimetic compounds, or TPM, Figure 2A), similar to the backbone of **13b**. Compared to **13b**, the (S)-γ-lactam ring at P1 position was expanded to the (S)-δ-lactam ring for better steric fitting with the S1 pocket (H163, E166, and H172). The S2 pocket exhibited hydrophobicity as the M49 and M165 residues stacking the isopropyl group we placed at P2 (Figure 2B). S3 and S4 constitute a long and narrow surface groove flanked by the P168 and Q189 residues. In the crystal structure, the interactions between Q189 and the **13b** pyridone ring are not optimal, resulting in the P3 groups being flipped away from the groove (Figure 2B and Figure 3C). Hence, we modified the pyridone ring with an amide bond and further diversified P3 with cyclohexane (TPM1), furan (TPM5), and vinyl benzene (TPM10, 16) groups (Figure 2C). For the control experiments, we also designed a tripeptidomimetic inhibitor TPM19 with less occupancy at the S3/S4 sub-pockets.

### 2.2. Biophysical Characterization of TPM–3CL^pro^ Interactions 

We synthesized five TPM inhibitors (i.e., TPM1, 5, 10, 16, and 19), determined their co-crystal structures with 3CL^Pro^ (Figure 3A, Table S1), and obtained substantial structural details (Figure 1C and Figure S2). The P1 (S)-δ-lactam ring formed hydrogen bond networks with the H163, E166, and H172 residues, while the P2 isopropyl group formed van der Waals contacts with M49 or M164 (Figure 3B). Compared to the rigidified conformation of the cyclopropyl group in **13b**, the isopropyl group of each TPM compound rotated to accommodate the subtle conformations of M49 and M164 (Figure 3B, right panel). More importantly, the P3 groups penetrated into the S3/S4 groove as intended (Figure 3C). A shorter P3 group in TMP19, by contrast, lead to outward-facing conformations, similar to that of **13b**. Hence, finely tuned P1–P3 substituents allowed us to examine a variety of designs with similar binding modes to 3CL^Pro^.

**Figure 4:**
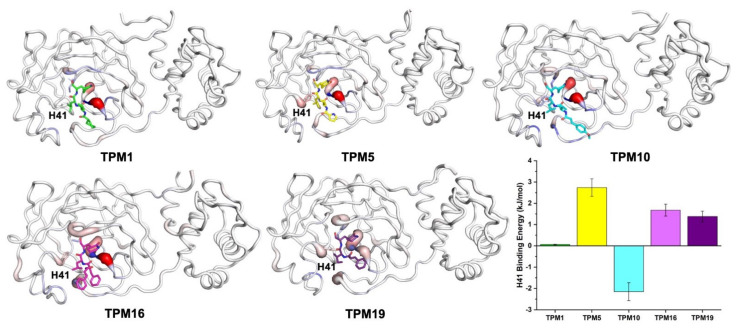
The interactions between TPMs and surrounding residues. The distribution of ΔG_bind_ on the 3CL^Pro^ residues are illustrated with grey (ΔG_bind_ ≈ 0), red (ΔG_bind_ > 0), and blue (ΔG_bind_ < 0) colors. The interaction energies between the H41 residue (shown as sticks) and TPMs are summarized as a histogram (average values of three independent trajectories, each of 1000 snapshots).

Based on the structural models, we simulated the dynamic processes of TPMs binding to 3CL^Pro^ using all-atom molecular dynamics (MD, Figure S3). All inhibitors stably resided within the pockets throughout the 100 ns trajectories. We calculated the binding free energies (ΔG_bind_) of each compound and mapped ΔG_bind_ to the residues surrounding the binding pocket (Figure 3D). In particular, we were interested in the contribution of the H41 residue to the binding energy (Figure 4 and Figure S4). TPM5 and TPM10 engaged with H41 with relatively strong interactions, while TPM1 exhibited little impact on H41. Comparing the backbones and binding modes of TPM1, 5, and 10, we postulated that the variety at P3 groups caused subtle changes in the interactions (attractive or repulsive) between H41 and TPMs.

**Figure 5:**
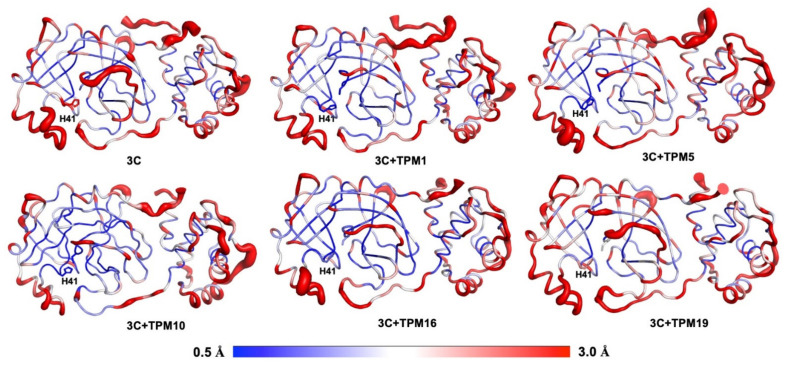
Conformational plasticity of 3CL^Pro^ and 3CL^Pro^–TPM complexes. The red bulges indicate flexible regions, while the blue colored regions were relatively rigid. TPMs 1, 5, and 10 significantly reduced the flexibility of the H41 residue.

We also analyzed the effects of TPM-binding on the kinetic properties of 3CL^Pro^. The RMSF calculations allowed us to compare the overall flexibilities of 3CL^Pro^ backbones with or without TPMs (Figure 5). Again, we focused on the H41 conformations. Notably, binding to TPMs generally rigidified H41 as evidenced by the reduced RMSF values compared to that of 3CL^Pro^-alone structures. The complex of 3CL^Pro^–TPM19, however, exhibited significant flexibility at the H41 position, indicating that the H41 residue was not locked in 3CL^Pro^–TPM19 or 3CL^Pro^-alone structures. As a positive control, we also simulated the dynamics of PF-07321332 and 3CL^Pro^ interactions and found the flexibility of H41 in the 3CL^Pro^–PF-07321332 complex to be similar to that of 3CL^Pro^–TPM19 (Figure S5).

### 2.3. Inhibitory Activity of TPMs against SARS-CoV-2 3CL^Pro^

**Figure 6:**
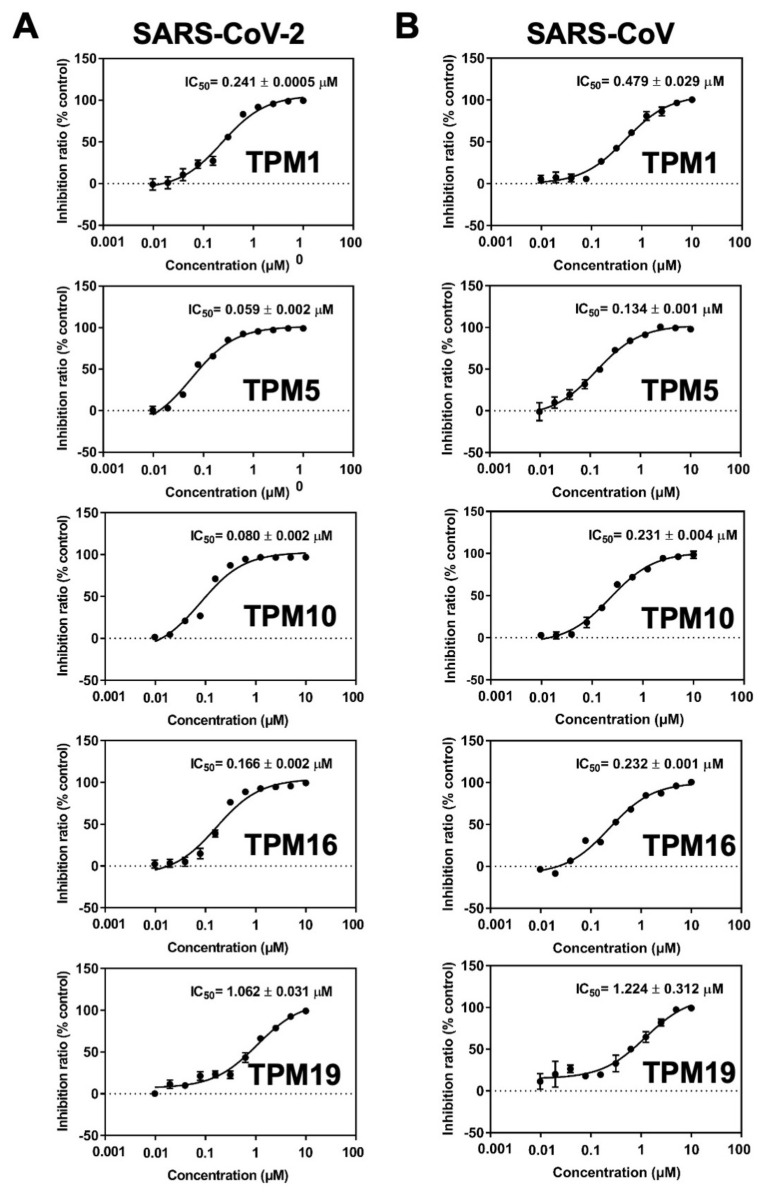
Dose–response curves of TPMs in the 3CL^Pro^ enzymatic activity assays. IC_50_ of inhibitors on SARS-CoV-2 (**A**) and SARS-CoV (**B**) 3CL^Pro^. From top to bottom: TPM1, TPM5, TPM10, TPM16, and TPM19. The TPM’s mediated inhibition was measured for both SARS-CoV-2 and SARS-CoV 3CL^Pro^. Bars depict the mean ± SE, *n* = 3.

We then measured the inhibitory activities of each TPM against SARS-CoV-2 3CL^Pro^ by a fluorescence resonance energy transfer (FRET)-based assay in which the MCA/DNP fluorophores labeled peptide (MCA-TSAVLQSGFRK(-DNP)M) was cleaved as the substrate. The half maximal inhibitory concentration (IC_50_) of 0.059–1.062 μM could be established for the TPMs. Notably, the tetrapeptidomimetic inhibitors moderately exceeded the lead compound **13b** (IC_50_ = 0.67 ± 0.18 μM) in terms of efficacy against 3CL^Pro^, while the tripeptidomimetic TPM19 did not (Figure 6A). Interestingly, the relative orders of the binding energies (TPM 10 > 1 > 5 > 16 > 19; Figure 3) was not the best predictor of the inhibitory activities (TPM 5 > 10 > 16 > 1 > 19). Instead, the binding energies between H41 and TPMs (Figure S6) or the flexibility of the H41 residue mostly agreed with the experimental data. For the peptidomimetic binders, the nature or the magnitude of their direct engagement with the catalytic residue H41 might determine their inhibitory effects on purified 3CL^Pro^.

To examine the potency of TPMs as broad-spectrum inhibitors, we also measured their IC_50_ against SARS-CoV 3CL^Pro^ using the same assay. We observed elevated IC_50_ (reduced efficacy) in the range of 0.134–1.224 μM (Figure 6B). However, the order of IC_50_ values among the five TPMs remained the same, likely due to the conserved proteolytic mechanism and high similarity of the binding pockets for the SARS-CoV and SARS-CoV-2 main protease.

### 2.4. The Ex-Vivo Anti-Viral Potencies of TPMs against SARS-CoV-2

**Figure 7:**
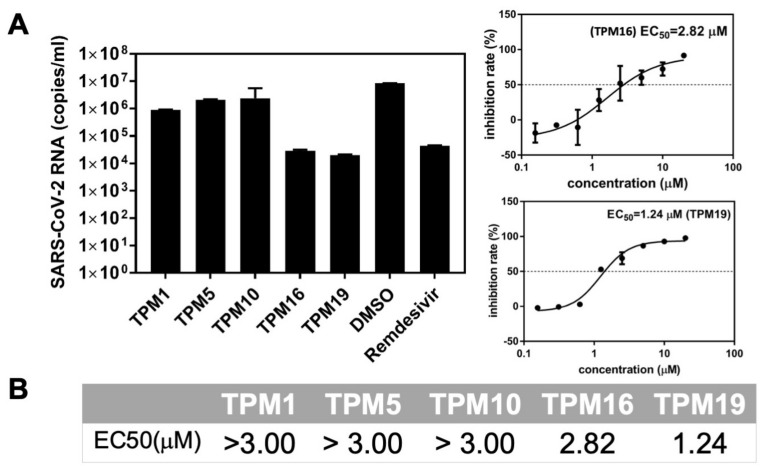
Anti-SARS-CoV-2 activity of the TPMs. (**A**) Left: The viral inhibitory activities were measured in Vero E6 cells at a final concentration of 10 μM. The cells were pre-treated with indicated compounds for 2 h, infected with SARS-CoV-2, and incubated for 2 days before the total RNA was isolated from cell lysates. The viral RNA content was analyzed with qPCR. DMSO was used as the vehicle control, and remdesivir was used as the positive control. Right: representative measurement of EC_50_ for the TPM ligands. (**B**) Summary of EC_50_ on Vero E6 cells.

We next evaluated the antiviral activity of TPMs at Vero E6 cell lines, which mimic the human lung environments, using SARS-CoV-2 infection assays. As the initial screen, TPM16 and TPM19 reduced the RNA loads of SARS-CoV-2 replicon by magnitudes of 10^3^ (relative level to the control experiment using DMSO), comparable to the effect of remdesivir, a viral RNA polymerase inhibitor (Figure 7A). TPM1, 5, and 10 all featured half maximal effective concentrations (EC_50_) above 3 μM, while TPM16 and 19 inhibited RNA replication with EC_50_ values of 2.82 and 1.24 μM, respectively. For comparison, the lead compound **13b** exhibited EC_50_ values of 4–5 μM for a different cell line (Calu-3) with a similar method of measuring viral RNA copies. The contrast between TPM5, 10, and 19 in terms of their IC_50_ and EC_50_ values highlighted the impact of the cellular environment on the development of clinically relevant candidates, because the uptake of our synthesized molecules by the cells will affect their effective concentrations. For a rough estimation, we collected the corresponding solvation energies of TPMs from the ΔG_bind_ calculations. Indeed, TPM16 and TPM19 featured relatively low energy costs in terms of solvation (187 and 132 kJ/moL, respectively; for the control, the solvation energy of PF-07321332 was 197 kJ/moL), while TPM1, 5, and 10 all featured solvation energies above 200 kJ/moL (Figure 3D). Thus, a balance of in vitro and ex vivo effects for TPM16 (IC_50_ = 0.16 μM, EC_50_ = 2.82 μM) likely reflect a balance between binding modes, impacts on the H41 conformations, and solvation. We also evaluated the cytotoxicity of TPMs with Vero E6 cell lines. TPMs did not affect the cellular viabilities in the range of concentrations we tested (the CC_50_ were evaluated to be >200 μM, Figure S7), suggesting their potencies in further development of antiviral drug candidates. 

## 3. Discussion and Conclusions

To meet the urgent need for developing antiviral drugs that can stall the rapid spread and constant mutation of SARS-CoV-2, researchers have screened the libraries of clinically safe drugs [20,21], designed structurally novel candidates with different strategies (peptidic or nonpeptidic covalent or noncovalent) [9,15]. Nonetheless, remdesivir, molnupiravir, and Paxlovid are the only FDA-approved small molecules for the treatment of COVID-19 thus far [22], with a few more candidates registered in phase II/III clinical trials [18,23]. This sharp contrast suggested substantial barriers in developing anti-SARS-CoV-2 drugs. The key unanswered question is how to establish a practical and easy-to-adapt way of evaluating the interplay between the viral targets (in current study, the 3CL^Pro^) and ligands [24,25]. In particular, the newly available drug Paxlovid (PF-07321332 and ritonavir) from Pfizer highlighted the potential of protease inhibitors and promoted the development of a diversity of peptidomimetic inhibitors (PF-07321332 is a tetrapeptidomimetic ligand). As evaluated by computational methods, PF-07321332 induced similar kinetic features compared to TPM16 (Figure S5), one of the best lead compounds found in the current study, suggesting the reliability of using simulated ΔG_bind_, RMSF, and solvation energies to guide drug discovery.

As a validated drug target among coronaviruses, 3CL^Pro^ offers several advantages, such as the breadth of action and dissimilarity to any human proteases [6,9]. We developed a computation-guide approach for in silico identification of beneficial modifications on 3CL^Pro^ inhibitors. The abilities of the designed tetrapeptidomimetic molecules to bind 3CL^Pro^, inhibit 3CL^Pro^-mediated proteolytic reactions, and reduce the virus loads were verified with combined structural, biophysical, and cellular experiments. For the discovery of guiding design principles, our data associated the simulated thermodynamic properties (ΔG_bind_ and RMSF) with the experimentally measured qualities (IC_50_ and EC_50_). The interactions between the ligands and the H41 residue of 3CL^Pro^ can be finely tuned by structural modifications for future development and optimization of a wide variety of antiviral protease inhibitors. 

## 4. Materials and Methods

### 4.1. Expression and Purification of the Recombinant Protein

The full-length gene encoding the SARS-CoV (GenBank: AY278488.2) and SARS-CoV-2 viruses (NC_045512.2) 3CL^Pro^ were optimized and synthesized for Escherichia coli expression by TsingKe Biological Technology. The plasmids were transformed into *Escherichia coli* BL21 (DE3) competent cells (TIANGEN, Hilden, Germany), and cultures were grown to OD_600_ = 0.8 in LB medium at 37 °C. Isopropyl-D-1-thiogalactopyranoside (IPTG) was added to a final concentration of 0.5 mM to induce the protein expression, and the cultures were grown at 16 °C overnight. Cells were harvested by centrifugation at 4500 rpm for 15 min, re-suspended, and homogenized in the lysis buffer containing 20 mM Tris-HCl (pH 8.0) and 300 mM NaCl using a low-temperature ultra-high-pressure cell disrupter (JNBIO, Guangzhou, China). Cell debris were removed by centrifugation at 18,000 rpm for 30 min. The resultant supernatants were added to Ni-NTA resin (GE Healthcare, Westborough, MA, USA). The nonspecific contaminants were removed by washing the resin with the buffer containing 50 mM imidazole. The target proteins were subsequently eluted with the buffer containing 20 mM Tris-HCl (pH 8.0), 300 mM NaCl, and 300 mM imidazole. Human rhinovirus 3C proteases were added to remove the C-terminal His tag. The target proteins were eluted and further purified by Superdex 200 (GE Healthcare, Westborough, MA, USA) and stored in 50 mM Tris-HCl (pH 7.3), 1 mM EDTA.

### 4.2. Synthesis of TPM Inhibitors

The synthesis of peptidomimetic inhibitors mainly followed the previously reported procedures [16,26]. Briefly, L-glutamic acid was Boc-protected using ditertbutyl dicarbonate and then the Cγ atom was alkylated and functionalized with a cyan group, followed by cyclization (P1 position). Then Boc-protected leucine amino acid were added with N-Ethyl-N’-(3-dimethylaminopropyl) carbodiimide hydrochloride to catalyze the formation of peptide bonds (P2 position), followed by reactions between cinnamic acid and the –NH_2_ (P4 position). The carboxyl group was converted to an aldehyde group with NaBH_4_ reduction and Dess–Martin Reagent. The reactants at each step were concentrated and purified on column chromatography.

### 4.3. In Vitro Enzymatic and Inhibition Assay

The fluorescent peptide MCA-AVLQSGFR-Lys(Dnp)-Lys-NH_2_ was employed as the substrate for the enzymatic and inhibition assay based on the fluorescence resonance energy transfer (FRET) effect. The half maximal inhibitory concentration (IC_50_) assay proceeded containing 0.2 μM SARS-CoV or SARS-CoV-2 virus 3CL^Pro^ in 20 mM Tris-HCl (pH 7.3) and 150 mM NaCl. The gradient diluted compounds were added to the buffer and incubated at 37 °C for 30 min, and 20 μM substrates were added into each well for 2 h, and OD intensities were read at λex = 320 nm and λem = 405 nm using a microplate reader (Thermo Scientific, Waltham, MA, USA).

### 4.4. Cytotoxicity

Cell viability assay was used to measure the inhibitors’ cytotoxicity following the CellTiter-Glo^®^ manufacturer’s instructions (Promega, Madison, WI, USA). Briefly, Vero E6 (3 × 10^4^ per well) cells were seeded in 96-well plates and cultured for 24 h. Serial dilutions of the compounds (0.78–200 µM in DMEM) were added and incubated for 48 h at 37 °C. Cells were incubated for 10 min with 100 µL of CellTiter-Glo^®^ reagent. The luminescence signals were determined using a microplate reader (Thermo Scientific, Waltham, MA, USA). The viability of cells treated with inhibitors were relativized to that of the non-treated cells.

### 4.5. Crystallization, Data Collection, and Structure Determination

The inhibitors and SARS-CoV-2 virus 3CL^Pro^ complexes were prepared by mixing the protease and the compounds at a 1:5 molar ratio and incubating at 4 °C for 2 h before setting up co-crystallization trials. The crystals were obtained by the hanging-drop vapor diffusion method at 16 °C. The crystals of complex appeared and reached their final size within 3 days in a well-solution containing 100 mM MES (pH 6.0), 3% DMSO, 1 mM DTT, and 2–8% polyethylene glycol (PEG) 6000.

For data collection, a single crystal was mounted on a nylon loop and was flash-cooled with a nitrogen gas stream at 100 K using 30% PEG400 as a cryo-protectant. Diffraction data were collected on BL19U1 at the Shanghai Synchrotron Radiation Facility (SSRF) at 100 K and at a wavelength of 0.97915 Å with an Pilatus3 6M image plate. Data were processed and scaled using the HKL3000 package [27]. The structures were determined using the molecular replacement (MR) method in the PHASER program [28] with the structure of apo SARS-CoV-2 3CL^Pro^ (PDB code: 6LU7) as the initial searching model. Manual model building and refinement were performed with the Coot and PHENIX programs following rigid body refinement, energy minimization, and individual B-factor refinement [29,30]. The final refinement statistics are summarized in Table S1.

### 4.6. Molecular Dynamics Simulations

The 100 ns all-atom simulations on the complex of 3CL^Pro^ and compounds were performed with the Gromacs 2019.6 package and CHARMM27 force field [31,32]. The initial poses of inhibitor-binding conformations were adopted from the crystal structures (PDB 7VH8 for the 3CL^Pro^ and PF07321332 structure). The system was solvated in a box (43.3 × 38.7 × 41.9 Å^3^) with TIP3P waters and 0.15 M NaCl with approximately 26,286 atoms in total. The topologies of TPM and PF07321332 inhibitors were generated by the SwissParam server [33]. First, the energy minimizations were performed to relieve unfavorable contacts, followed by 10 ns equilibration steps. Then, the simulations were performed at 300 K (velocity-rescale thermostat) and constant pressure (1 bar, Parrinello–Rahman NPT ensemble). The nonbonded interaction cut-off for electrostatics calculations was set at 10 Å and the particle mesh Ewald (PME) method was used for calculation of long-range electrostatic interactions. LINCS constraints were applied to H bonds and the time step was 2 fs. For each compound, three independent simulations were performed. Throughout the trajectories, the representative binding conformations were clustered based on their structural similarities. After reaching equilibrium, g_mmpbsa modules were evoked for the calculation of binding energies between the inhibitors and protein using 1000 frames, on average, wherein the energy could be decomposed on a per residue basis [34]. 

## Data Availability

The coordinates and structural models of 3CL^Pro^–TPM complexes have been deposited at the Protein Data Bank (TPM1: 7WO1, TPM5: 7WO2, TPM10: 7WO3, TPM16: 7WOH, and TPM19: 7WOF). The chemical, plasmids, and data set used in this study are available upon request from the corresponding authors.

## Supplementary Materials

Supplementary Materials are available online at https://www.mdpi.com/article/10.3390/ijms23042392/s1.
