# Supplementary material for: Discovery of SARS-CoV-2 3CLPro Peptidomimetic Inhibitors through the Catalytic Dyad Histidine-Specific Protein–Ligand Interactions
Source: Int J Mol Sci. 2022 Feb 21;23(4):2392. doi: 10.3390/ijms23042392 (PMC8878928; doi:10.3390/ijms23042392)
Supplement: Supplementary file 1 [file ijms-23-02392-s001.zip › ijms-1601706-supplementary.pdf]

Supplementary Table S1. Crystallography data collection and refinement statistics

Parameters	TPM1 (PDB code:7WO1)
X-ray Source	BL19U1
Wavelength (Å)	0.97915
Space group	$P2_12_12$
Unit cell parameters (Å)	*a*=45.0, *b*=62.4, *c*=104.6
Resolution range (Å)	50.0-2.15 (2.20-2.15)*
Unique reflections	16,430 (419)
Completeness (%)	97.0 (52.7)
Redundancy	7.1 (2.6)
I/σ(I)	23.6 (2.3)
R_{merge} (%)	9.3 (33.9)
R_{meas} (%)	10.0 (41.2)
R_{pim} (%)	3.5 (22.8)
$CC_{1/2}$	0.998 (0.811)
**Refinement statistics**	
Resolution range (Å)	27.59-2.15 (2.23-2.15)
Reflections used in refinement	16,392 (1,469)
Reflections used for R-free	1,639 (147)
R_{work} (%)	17.7 (21.9)
R_{free} (%)	22.3 (24.7)
Number of non-hydrogen atoms	2,538
Protein	2,367
Solvent	136
Ligand	35
Average B-factors	29.8
Protein	29.3
Solvent	37.1
Ligand	35.0
r.m.s. deviations	
Bond lengths (Å)	0.002
Bond angles (°)	0.45
Ramachandran	
Favored (%)	97.7
Allowed (%)	2.3
Outliers (%)	0.0

*Numbers in the brackets are for the highest resolution shell.

Parameters	TPM5 (PDB code: 7WO2)
X-ray Source	BL19U1
Wavelength (Å)	0.97915
Space group	$P2_12_12$
Unit cell parameters (Å)	*a*=45.0, *b*=62.9, *c*=106.4
Resolution range (Å)	50.0-1.96 (1.99-1.96)*
Unique reflections	22,409 (1,579)
Completeness (%)	100.0 (100.0)
Redundancy	10.5 (7.6)
I/σ(I)	53.7 (17.8)
R_{merge} (%)	9.1 (21.3)
R_{meas} (%)	9.6 (22.8)
R_{pim} (%)	2.9 (8.0)
$CC_{1/2}$	0.998 (0.989)
**Refinement statistics**	
Resolution range (Å)	23.53-1.96 (2.03-1.96)
Reflections used in refinement	22,367 (2,175)
Reflections used for R-free	1,190 (110)
R_{work} (%)	18.2 (18.4)
R_{free} (%)	21.8 (22.5)
Number of non-hydrogen atoms	2,647
Protein	2,329
Solvent	284
Ligand	34
Average B-factors	18.2
Protein	17.1
Solvent	27.0
Ligand	18.3
r.m.s. deviations	
Bond lengths (Å)	0.007
Bond angles (°)	1.0
Ramachandran	
Favored (%)	98.0
Allowed (%)	2.0
Outliers (%)	0.0

*Numbers in the brackets are for the highest resolution shell.

Parameters	TPM10 (PDB code: 7WO3)
X-ray Source	BL19U1
Wavelength (Å)	0.97915
Space group	$P2_12_12$
Unit cell parameters (Å)	*a*=45.2, *b*=62.9, *c*=105.0
Resolution range (Å)	50.0-2.01 (2.03-2.01)*
Unique reflections	20,375 (947)
Completeness (%)	98.3 (92.5)
Redundancy	7.4 (4.9)
I/σ(I)	10.4 (1.6)
R_{merge} (%)	19.2 (98.8)
R_{meas} (%)	20.8 (109.7)
R_{pim} (%)	7.7 (45.7)
$CC_{1/2}$	0.991 (0.476)
**Refinement statistics**	
Resolution range (Å)	31.45-2.01 (2.08-2.01)
Reflections used in refinement	18,827 (1,360)
Reflections used for R-free	922 (62)
R_{work} (%)	19.6 (22.5)
R_{free} (%)	24.3 (29.6)
Number of non-hydrogen atoms	2,511
Protein	2,340
Solvent	132
Ligand	39
Average B-factors	29.0
Protein	28.6
Solvent	34.8
Ligand	35.7
r.m.s. deviations	
Bond lengths (Å)	0.007
Bond angles (°)	0.9
Ramachandran	
Favored (%)	96.4
Allowed (%)	3.7
Outliers (%)	0.0

*Numbers in the brackets are for the highest resolution shell.

Parameters	TPM16 (PDB code: 7WOH)
X-ray Source	BL19U1
Wavelength (Å)	0.97915
Space group	*P*1
Unit cell parameters (Å)	*a*=51.8, *b*=63.2, *c*=63.1
	α=78.5, *β*=71.4, *γ*=71.5
Resolution range (Å)	19.85-1.72 (1.78-1.72)*
Unique reflections	68,709 (5,575)
Completeness (%)	89.6 (72.7)
Redundancy	3.6 (3.2)
I/σ(I)	40.6 (2.9)
R_{merge} (%)	2.4 (13.3)
R_{meas} (%)	2.8 (15.7)
R_{pim} (%)	1.4 (8.3)
$CC_{1/2}$	1.000 (0.990)
**Refinement statistics**	
Resolution range (Å)	19.48-1.72 (1.78-1.72)
Reflections used in refinement	68,520 (6,011)
Reflections used for R-free	3,522 (277)
R_{work} (%)	17.9 (22.2)
R_{free} (%)	19.8 (29.0)
Number of non-hydrogen atoms	5,187
Protein	4,680
Solvent	425
Ligand	82
Average B-factors	34.5
Protein	33.6
Solvent	41.1
Ligand	44.8
r.m.s. deviations	
Bond lengths (Å)	0.009
Bond angles (°)	1.2
Ramachandran	
Favored (%)	96.5
Allowed (%)	3.5
Outliers (%)	0.0

*Numbers in the brackets are for the highest resolution shell.

Parameters	TPM19 (PDB code:7WOF)
X-ray Source	BL19U1
Wavelength (Å)	0.97915
Space group	*C*121
Unit cell parameters (Å)	*a*=97.4, *b*=79.5, *c*=51.7<br>*α*=90.0, *β*= 114.0, *γ*=90.0
Resolution range (Å)	19.88-1.72 (1.78-1.72)*
Unique reflections	3,7643 (3,254)
Completeness (%)	96.5 (98.4)
Redundancy	6.6 (5.6)
I/σ(I)	27.5 (5.4)
R_{merge} (%)	3.5 (22.8)
R_{meas} (%)	3.8 (25.1)
R_{pim} (%)	1.4 (10.2)
$CC_{1/2}$	0.999 (0.977)
**Refinement statistics**	
Resolution range (Å)	19.77-1.72 (1.78-1.72)
Reflections used in refinement	37,279 (3,434)
Reflections used for R-free	1,762 (162)
R_{work} (%)	19.8 (26.3)
R_{free} (%)	20.4 (29.6)
Number of non-hydrogen atoms	2,627
Protein	2,340
Solvent	257
Ligand	30
Average B-factors	31.0
Protein	30.4
Solvent	35.2
Ligand	34.6
r.m.s. deviations	
Bond lengths (Å)	0.007
Bond angles (°)	0.9
Ramachandran	
Favored (%)	97.0
Allowed (%)	3.0
Outliers (%)	0.0

*Numbers in the brackets are for the highest resolution shell.

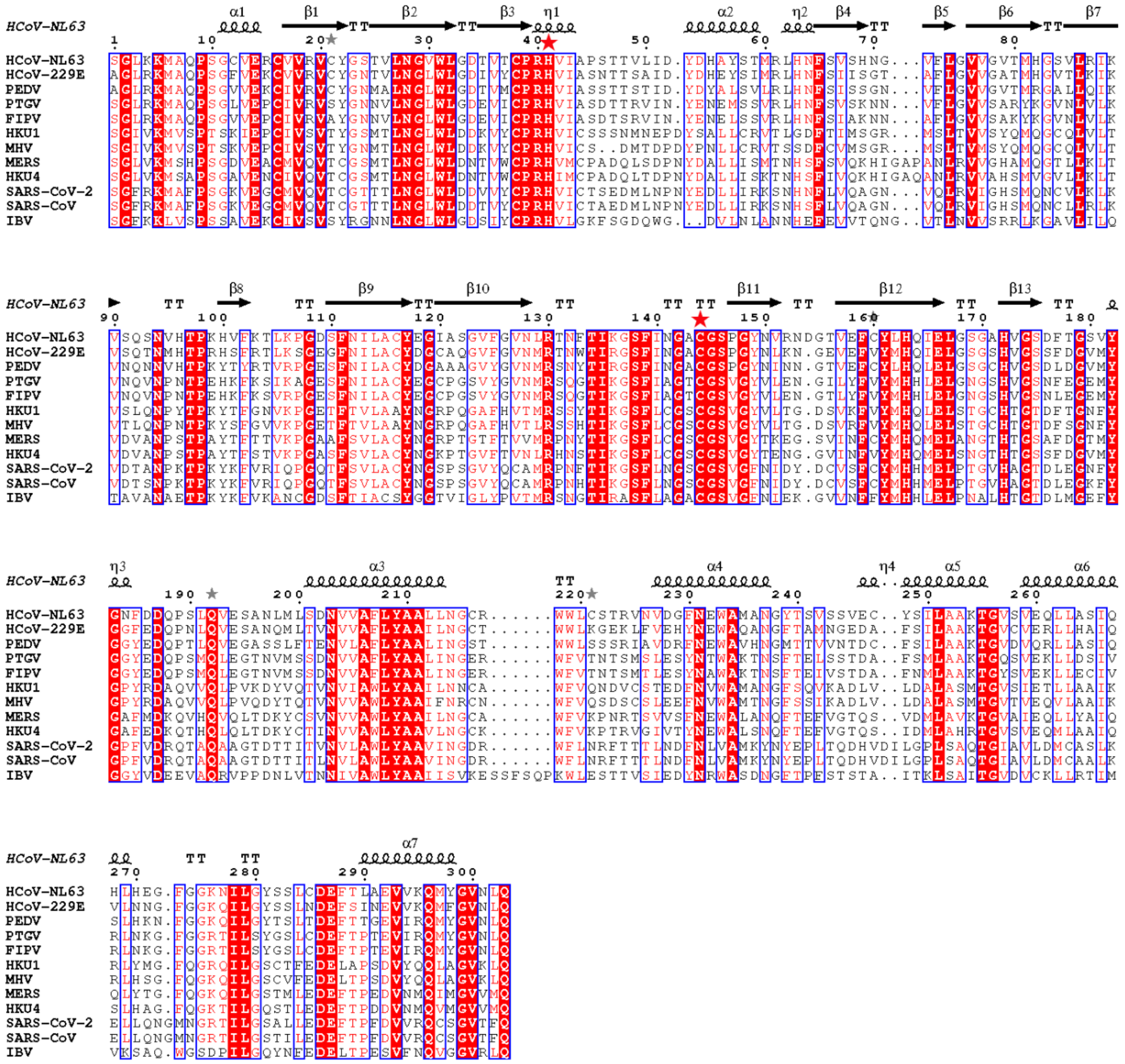

Figure S1. Multi-sequence alignment of 3CL^{Pro} from twelve members of coronaviridae family. Conserved residues were marked with blue frames and identical residues were highlighted in red. The H41 and C145 catalytic dyad was labeled with red stars on top of the column.

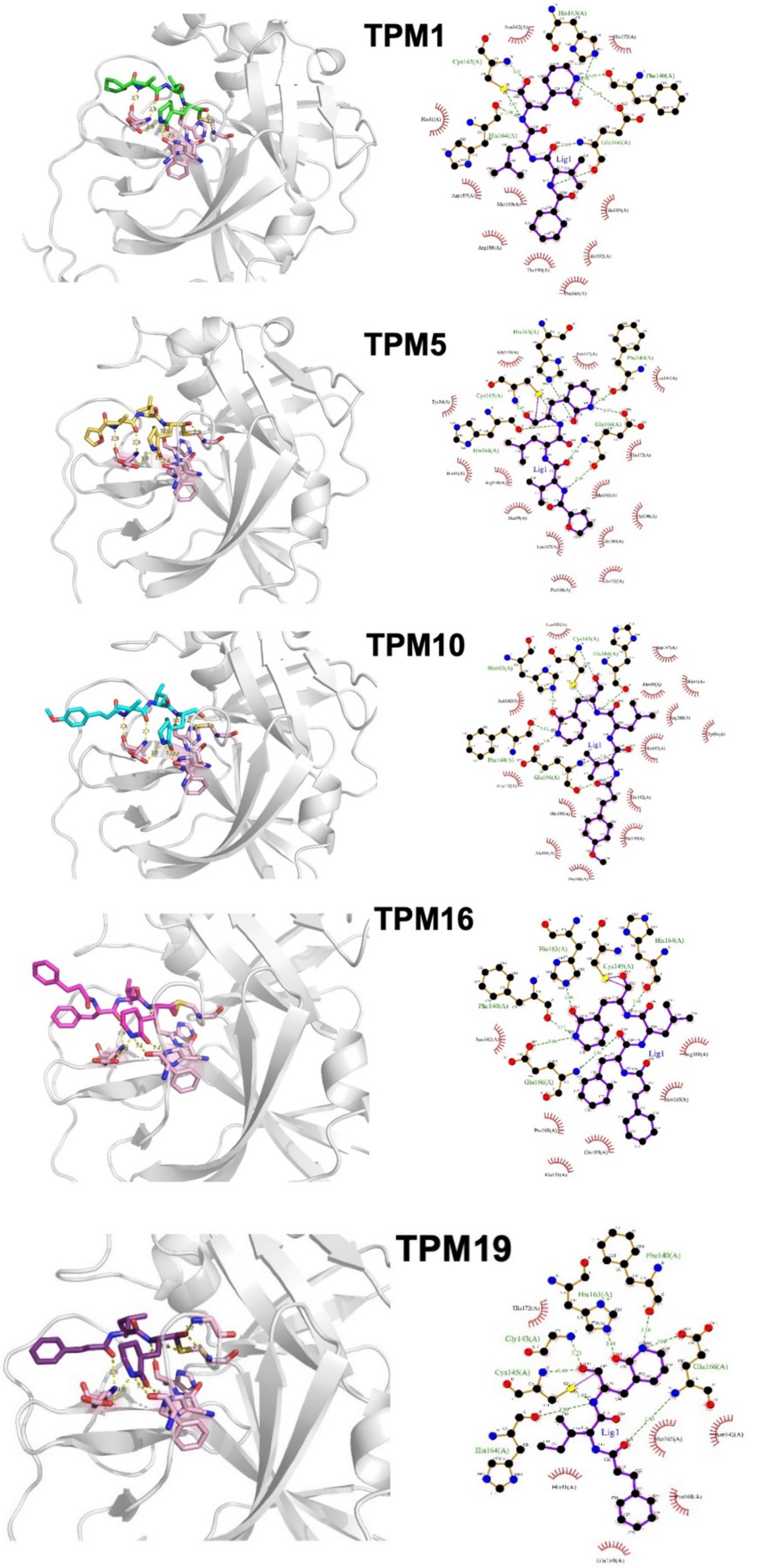

Figure S2. Key residues surrounding the TPM molecules were indicated as pink sticks.

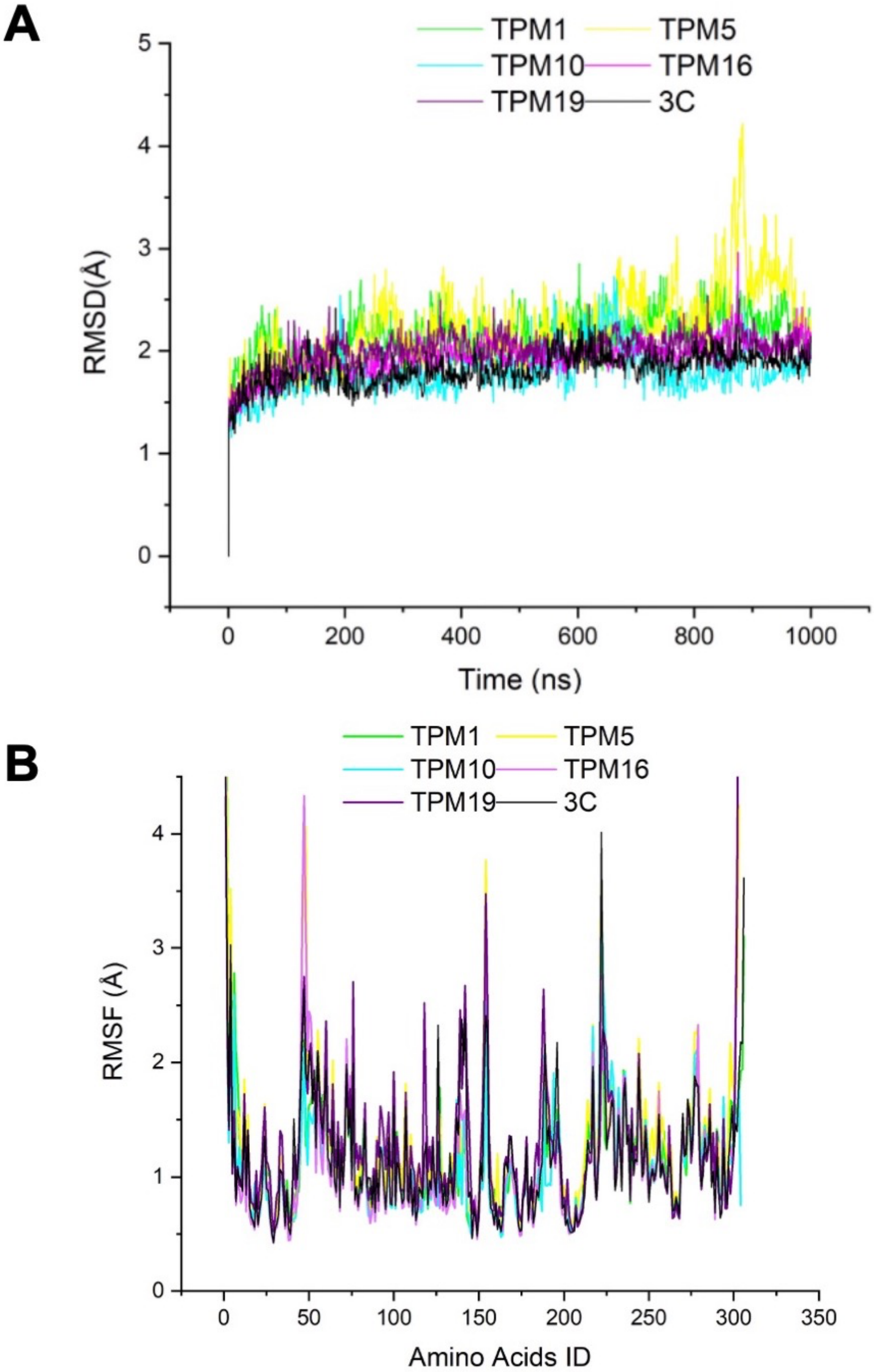

Figure S3. Kinetic parameters of the 3CL^{pro} – TPM simulations. (A) The root-mean-square deviation (RMSD) of representative 100 ns trajectories. (B) The root mean square fluctuation (RMSF) measurements of the displacement of each residue in 3CL^{pro}, related to Figure 5 of the main text.

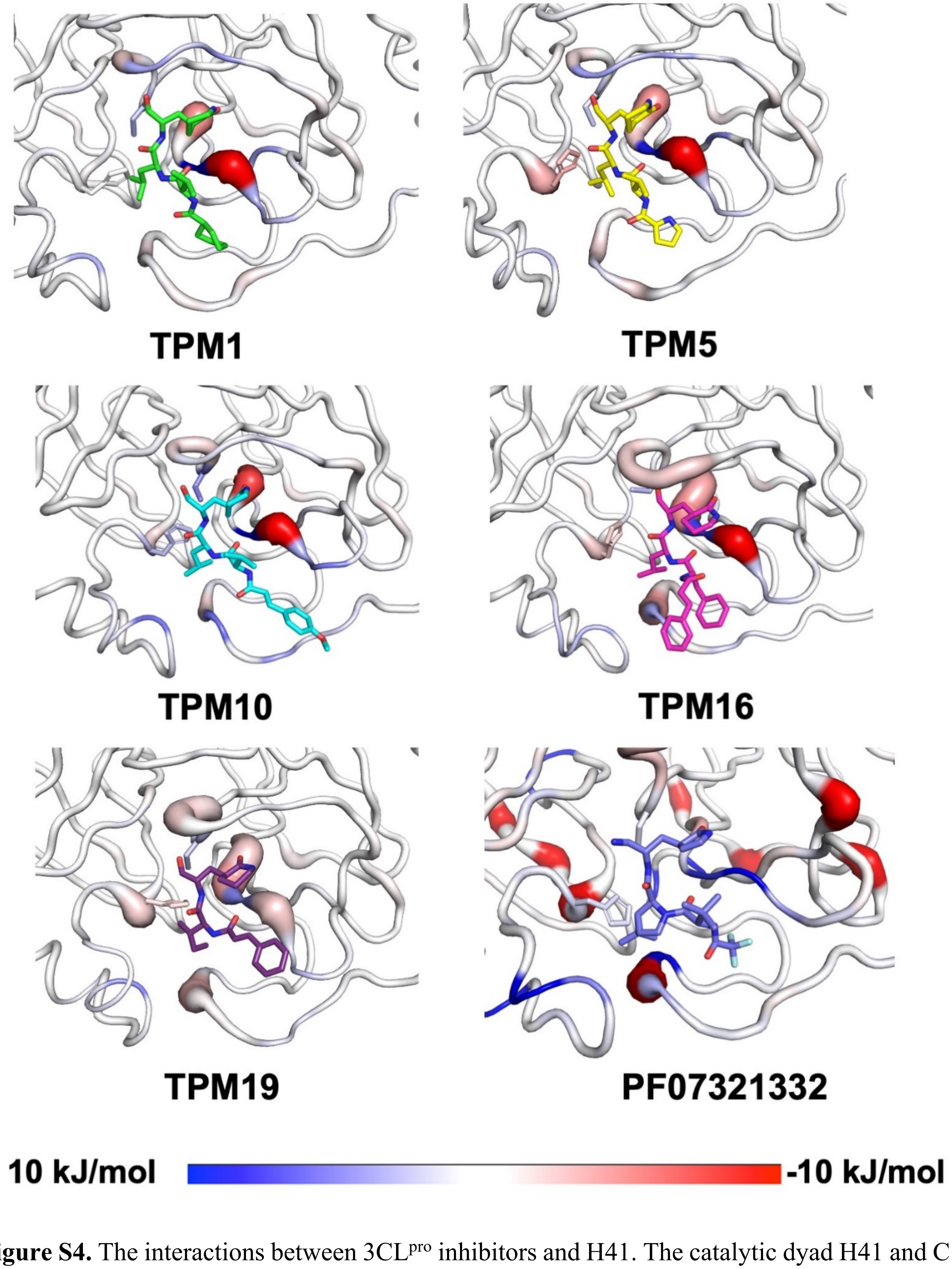

Figure S4. The interactions between 3CL^{pro} inhibitors and H41. The catalytic dyad H41 and C145 residues were shown as sticks and colored by the magnitude of their interactions with each ligand, related to Figure 4 of the main text.

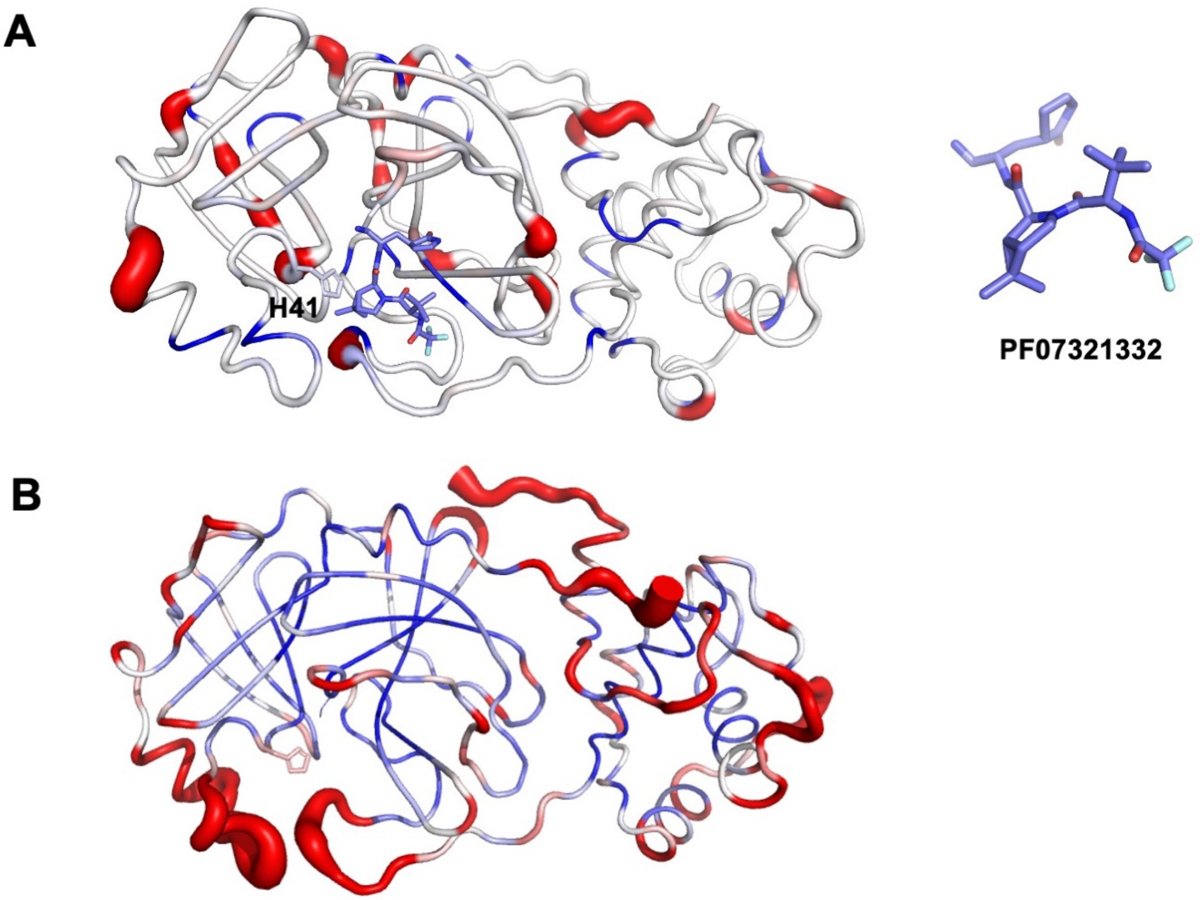

Figure S5. Simulations on the 3CL^{pro} – PF07321332 interactions. (A) The binding energies between PF07321332 and the 3CL^{Pro} residues were illustrated with grey ($\Delta G_{bind} \approx 0$), red ($\Delta G_{bind} > 0$), blue ($\Delta G_{bind} < 0$) colors. The H41 residue was illustrated as sticks and the ΔG_{bind} of H41 to PF07321332 was -1.29 kJ/mol. Notably, the protein-ligand interactions of 3CL^{pro} – TPMs were largely confined to the reactive center (surrounding C145 and H41, Figure 4 in the main text), while the residue level interactions to PF07321332 were dispersed on the 3CL^{pro} scaffold, indicating PF07321332 induced stronger interactions not only in the binding pocket but also in remote regions. (B) Conformational plasticity of the 3CL^{Pro} – PF07321332 complex. The H41 residue was illustrated as sticks and its red color indicated residue flexibility comparable to that of 3CL^{Pro} – TPM19 (illustrated in Figure 5 of the main text).

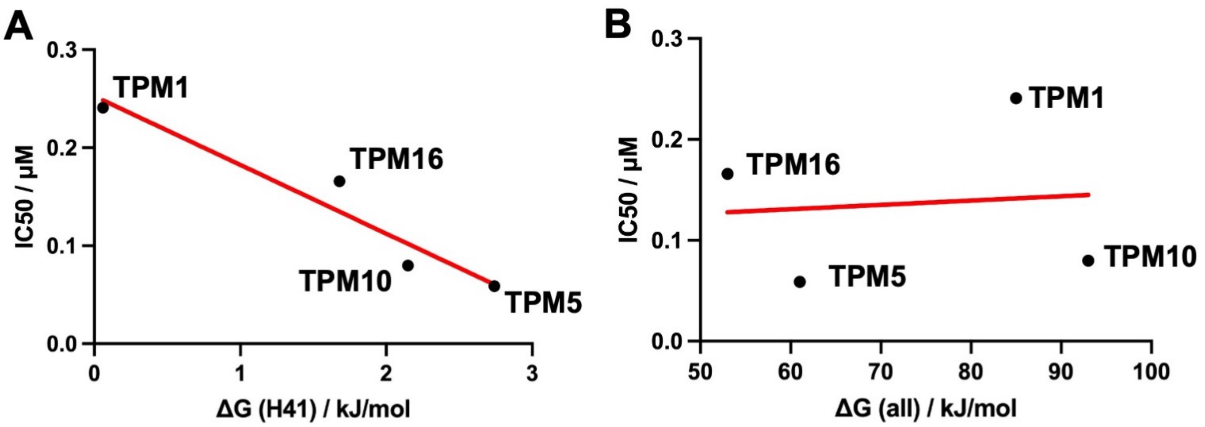

Figure S6. Fitting between experimentally measured IC_{50} and computationally predicted ΔG_{bind}: (A) H41 residue – TPM interaction energies, (B) 3CLpro – TPM interaction energies. The data of tetrapeptidomimetic inhibitors were plotted and the X axis demonstrated absolute values of ΔG_{bind} (kJ/mol).

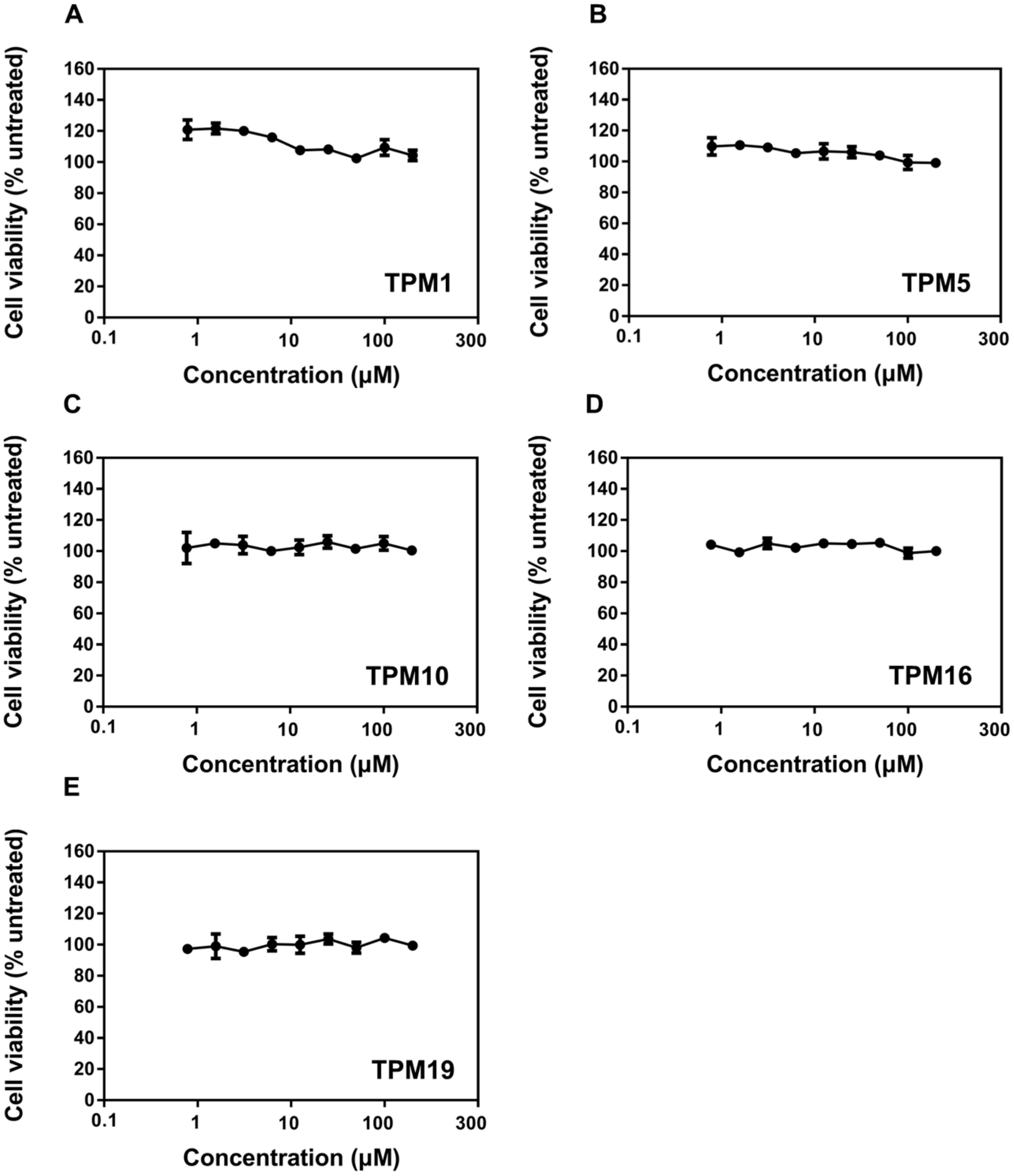

Figure S7 The cell viabilities after TPM compounds treatment were measured by series dilution on Vero E6 cells.
